# Transient Interferon-Driven Natural Killer Cell Activation in Acute Hepatitis C

**DOI:** 10.1093/infdis/jiaf654

**Published:** 2025-12-29

**Authors:** Benedikt Strunz, Qiuyao Zhan, Tanvi Khera, Julia Hengst, Marija Jankovic, Katja Deterding, Annika Niehrs, Markus Cornberg, Cheng-Jian Xu, Heiner Wedemeyer, Niklas K Björkström, the HepNet Acute HCV IV Study Group, Christoph D Spinner, Eckart Schott, Tania M Welzel, Guido Gerken, Hartwig Klinker, Ulrich Spengler, Johannes Wiegand, Julian Schulze zur Wiesch, Anita Pathil, Andreas Umgelter, Caroline Zöllner, Stefan Zeuzem, Armin Papkalla, Kristina Weber, Svenja Hardtke, Heiko von der Leyen, Armin Koch, Dorothee von Witzendorff, Michael P Manns, Kerstin Port, Bernhard Schlevogt, Marc Ringelhan, Ulrich Mayr, Judith Schrewe, Katharina Sosnowsky, Christoph Jochum, Gudrun Hilgard, Petra Schulze, Susanne Wiebecke, Ji-Eun Lee, Peter Hoffmann

**Affiliations:** Center for Infectious Medicine, Department of Medicine Huddinge, Karolinska Institutet, Karolinska University Hospital, Stockholm, Sweden; Centre for Individualised Infection Medicine (CIIM), Helmholtz-Centre for Infection Research (HZI) and Hannover Medical School, Hannover, Germany; TWINCORE, Centre for Experimental and Clinical Infection Research, Helmholtz-Centre for Infection Research (HZI) and Hannover Medical School, Hannover, Germany; TWINCORE, Centre for Experimental and Clinical Infection Research, Helmholtz-Centre for Infection Research (HZI) and Hannover Medical School, Hannover, Germany; TWINCORE, Centre for Experimental and Clinical Infection Research, Helmholtz-Centre for Infection Research (HZI) and Hannover Medical School, Hannover, Germany; Center for Infectious Medicine, Department of Medicine Huddinge, Karolinska Institutet, Karolinska University Hospital, Stockholm, Sweden; Department of Gastroenterology, Hepatology and Endocrinology, Hannover Medical School, Hannover, Germany; Center for Infectious Medicine, Department of Medicine Huddinge, Karolinska Institutet, Karolinska University Hospital, Stockholm, Sweden; Centre for Individualised Infection Medicine (CIIM), Helmholtz-Centre for Infection Research (HZI) and Hannover Medical School, Hannover, Germany; TWINCORE, Centre for Experimental and Clinical Infection Research, Helmholtz-Centre for Infection Research (HZI) and Hannover Medical School, Hannover, Germany; Department of Gastroenterology, Hepatology and Endocrinology, Hannover Medical School, Hannover, Germany; German Center for Infection Research (DZIF), Partner Site Hannover-Braunschweig, Braunschweig, Germany; Cluster of Excellence RESIST (EXC2155), Hannover Medical School, Hannover, Germany; HepNet Study House of the German Liver Foundation, Hannover, Germany; Centre for Individualised Infection Medicine (CIIM), Helmholtz-Centre for Infection Research (HZI) and Hannover Medical School, Hannover, Germany; TWINCORE, Centre for Experimental and Clinical Infection Research, Helmholtz-Centre for Infection Research (HZI) and Hannover Medical School, Hannover, Germany; Department of Gastroenterology, Hepatology and Endocrinology, Hannover Medical School, Hannover, Germany; German Center for Infection Research (DZIF), Partner Site Hannover-Braunschweig, Braunschweig, Germany; Cluster of Excellence RESIST (EXC2155), Hannover Medical School, Hannover, Germany; HepNet Study House of the German Liver Foundation, Hannover, Germany; Center for Infectious Medicine, Department of Medicine Huddinge, Karolinska Institutet, Karolinska University Hospital, Stockholm, Sweden

**Keywords:** acute hepatitis C, NK cells, single-cell RNA sequencing

## Abstract

Acute infection with hepatitis C virus (HCV) is a rare event that can be treated successfully with direct-acting antivirals (DAAs). As natural killer (NK) cells play an important role during the natural course of acute HCV, we assessed the NK cell compartment via flow cytometry and single-cell sequencing in longitudinally sampled patients with acute HCV and compared this to healthy controls and patients with chronic HCV. At the transcriptomic level, we identified a subset of highly activated NK cells with a robust type I interferon imprint. While the population of activated NK cells vanished after DAA-mediated cure, a long-term phenotypic imprint of infection was observed in comparison to healthy controls. Collectively, these data suggest an interferon-driven rise of an activated NK cell population during acute hepatitis C that is largely restored upon viral clearance. This study provides insights into the immunological basis for successful antiviral response to hepatitis C.

## Teaser

Natural killer cells display an interferon-mediated activation during the acute symptomatic infection with hepatitis C virus that is restored after viral clearance.

Hepatitis C virus (HCV) infection rarely manifests as acute symptomatic infection [1]. Typically, it progresses to a chronic infection in most patients and, if untreated, leads to liver cirrhosis with consecutively high morbidity/mortality [1, 2]. However, when identified, acute symptomatic hepatitis C was previously treated with an interferon (IFN)–based therapy [3] but now is treated successfully with direct-acting antivirals (DAAs) [4]. While the function and dysfunction of the immune system have been extensively studied in chronic HCV infection [5], immune functions during viral clearance in the acute symptomatic setting remain to be elucidated.

Natural killer (NK) cells are innate lymphocytes that are enriched in the liver [6] and known to participate in the response to viral infections [7]. As such they likely contribute to the immune response in acute hepatitis C. Indeed, on the genetic level, certain combinations of inhibitory NK cell receptors (killer cell immunoglobulin-like receptor) and their ligands strongly associate with viral clearance of acute HCV [8]. Yet, once the HCV infection becomes chronic, NK cell function is hampered and both cytokine production and cytotoxicity can be affected [9]. Even though NK cell alterations in acute HCV have been studied to some extent [10, 11], their dynamic profile during DAA-mediated clearance has not been investigated in detail.

A key cytokine family involved in control of HCV infection is type I IFNs [12], which trigger transcription of a plethora of IFN-stimulated genes (ISGs) [13]. The impact of type I IFNs on anti-HCV immunity is evident by the treatment success of pegylated IFN-α in the pre-DAA era [14]. Furthermore, it has been shown that the NK cell compartment is altered in response to IFN treatment [15]. However, if and how the natural type I IFN response affects NK cells in acute HCV is unknown.

In the present study we analyzed the peripheral blood NK cell compartment in patients with acute hepatitis C before, during, and after DAA-mediated viral clearance. We describe the presence of a strongly activated NK cell subpopulation displaying an ISG signature that normalized after viral cure. This stands in contrast to chronic HCV, in which only minor alterations occurred during viral clearance. To conclude, here we report dynamic and largely reversible alterations of the NK cell population that associate with acute HCV.

## MATERIALS AND METHODS

### Patient and Control Cohorts

In this study, 15 patients with acute symptomatic infection with HCV genotype 1 were included and treated with a 6-week course of ledipasvir/sofosbuvir (LDV/SOF) as previously described through the HepNet clinical trial (NCT02309918) [4]. Furthermore, 12 patients with chronic HCV infection sampled before (baseline) and 24 weeks after treatment with LDV/SOF and 20 healthy controls were recruited for comparison. See Supplementary Table 1 for clinical information. For detailed methods, please see the Supplementary Materials.

## RESULTS

### Single-Cell Analysis of Peripheral Blood Mononuclear Cells Identifies a Subset of Highly Activated NK Cells During Acute HCV Infection

**Figure 1.**
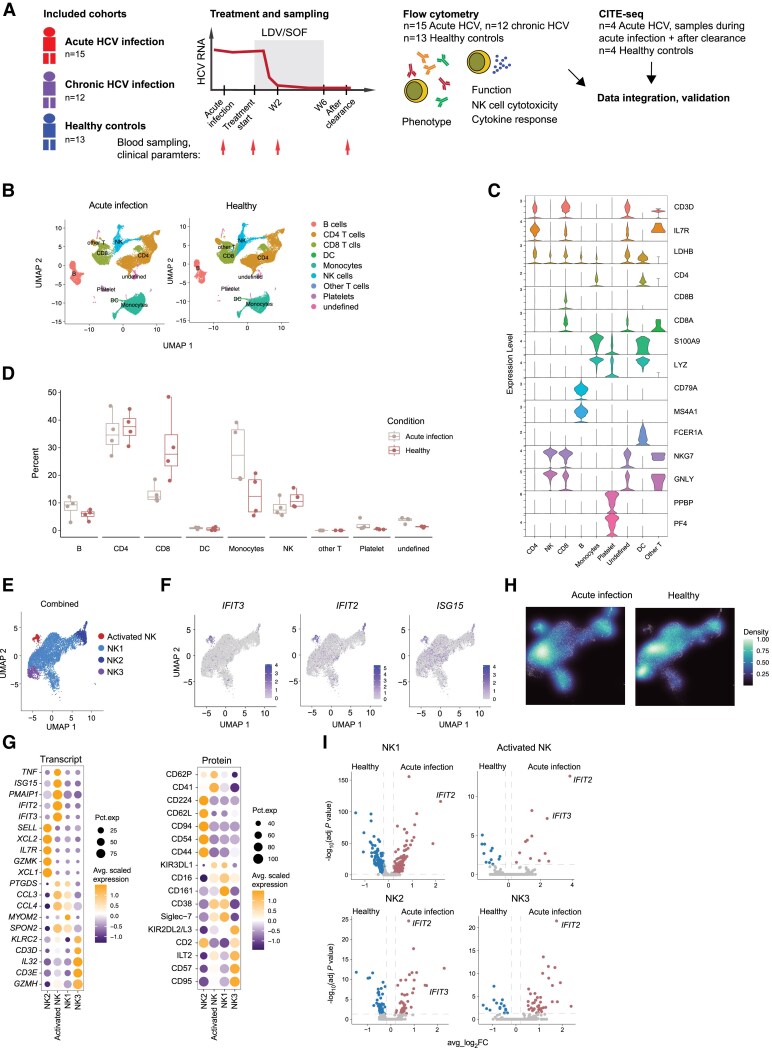
A cluster of interferon-activated NK cells are present in acute HCV infection. *A*, Overview of study design. *B*, UMAP plot displaying scRNA-seq profiles of immune cells from patients with acute HCV (left, n = 4) and healthy controls (right, n = 4). Each dot represents a single cell, and colors indicate cell-type identity. *C*, Violin plots show gene expression levels of representative marker genes across cell types. *D*, Comparison of the immune cell proportions between patients with acute HCV and healthy controls. *E*, Subclustering UMAP plot of NK cells, including activated NK cells and NK1–NK3. *F*, Interferon markers overlaid on UMAP embedding, including *IFIT2*, *IFIT3*, and *ISG15*. *G*, Dot plot showing the expression levels of the top 5 genes and proteins in each NK subcluster. *H*, UMAP density plots illustrating the distribution of cells from patients with acute HCV and healthy controls. *I*, Volcano plots displaying DEGs in each NK subcluster. Red dots indicate significantly differentially expressed genes (FDR <0.05 and avg_log_2_FC >0.2). Blue dots represent genes (FDR <0.05 and avg_log_2_FC < −0.2). Abbreviations: CITE-seq, cellular indexing of transcriptome and epitopes by sequencing; DC, dendritic cell; DEG, differentially expressed gene; FC, fold change; FDR, false discovery rate; HCV, hepatitis C virus; LDV/SOF, ledipasvir/sofosbuvir; NK, natural killer; scRNA-seq, single-cell RNA sequencing; UMAP, uniform manifold approximation and projection.

NK cells are of importance for the clearance of HCV infection [8]. To study the NK cell compartment in the rare manifestation of acute symptomatic HCV, 15 patients with acute HCV participating in the HepNet Acute HCV IV trial [4] were sampled before, during, and after DAA treatment and compared to healthy controls and patients with chronic HCV (Figure 1*A*). All patients included in this study cleared the virus during treatment [4]. This setting offered the unique possibility to investigate the NK cell compartment in acute compared to chronic infection as well as in relation to the impact of virological cure. With cellular indexing of transcriptome and epitopes by sequencing (CITE-seq) and multiparameter flow cytometry, we were able to longitudinally study the transcriptome, phenotype, and function of NK cells. As a starting point, NK cells and the major immune cell subtypes could readily be identified in the single-cell RNA sequencing (scRNA-seq) data (Figure 1*B* and 1*C*, Supplementary Figure 1*A–C*). No differences in regard to immune cell frequencies were observed (Figure 1*D*). To study the NK cell compartment in detail, we first stratified for the recently defined human NK cell subsets [16]. Of interest, we could identify not only the 3 previously described NK cell clusters (NK1–NK3) but also 1 additional cluster (Figure 1*E*). This additional cluster consisted of NK cells displaying a ISG and activation signature, as indicated by high expression of the transcripts *IFIT2*, *IFIT3*, and *ISG15* as well as elevated functional markers such as *CCL3*, *CCL4*, and *TNF* (Figure 1*F* and 1*G*). Moreover, these activated NK cells were enriched in patients with acute HCV compared to healthy controls (Figure 1*F* and 1*H*). However, when comparing each NK cell subset separately to healthy controls, an upregulation of ISGs, such as *IFIT2*, was observed in the NK1, 2, and 3 subsets (Figure 1*I*).

Collectively, we identified a robust activation of NK cells and the presence of a cluster of activated NK cells marked transcriptomically by an ISG signature.

### Phenotypic and Functional Alteration of NK Cells in Acute HCV Infection

**Figure 2.**
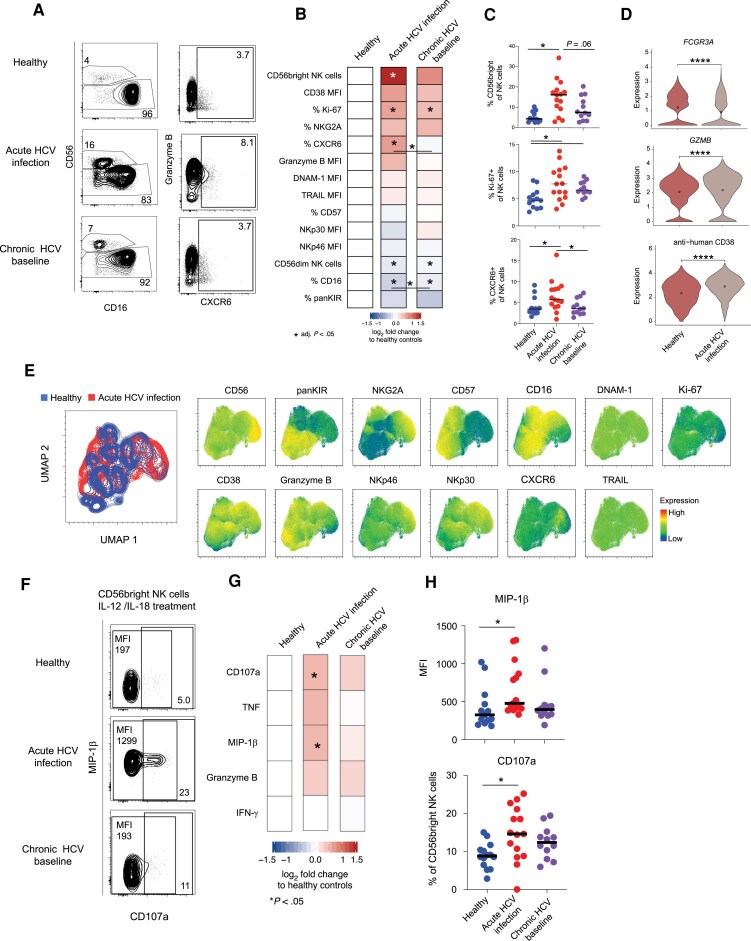
Phenotypical identification of a CXCR6^+^ NK cell population and NK cell function in acute HCV. *A–C*, Representative flow cytometry plots (*A*), combined analysis (*B*), and representative scatter plots (*C*) for NK cells in healthy individuals (n = 13), patients with acute HCV (n = 15), and patients with chronic HCV (n = 12). *D*, Violin plots comparing the expression of *FCGR3A*, *GZMB*, and CD38 in cells from healthy individuals and patients with acute HCV; diamond-shaped markers represent mean expression. Significance was assessed by the Wilcoxon test with Bonferroni correction. ****FDR <0.0001. *E*, UMAP analysis of pooled samples from downsampled total NK cells (healthy, n = 13; acute HCV, n = 14). Included for UMAP generation were the displayed phenotypic markers. *F–H*, Functional assessment of CD56^bright^ NK cells after IL-12/IL-18 stimulation. Representative flow cytometry stainings (*F*) and scatter plot (*H*) of MIP-1β and CD107a. *G*, Summary of data from healthy individuals (n = 13), patients with acute HCV (n = 13), and patients with chronic HCV (n = 12). Statistical significance was tested with Kruskal–Wallis test followed by Dunn multiple comparisons test. *P < .05. Abbreviations: FDR, false discovery rate; HCV, hepatitis C virus; IFN-γ, interferon gamma; IL, interleukin; MFI, median fluorescence intensity; MIP-1β, macrophage inflammatory protein 1β; NK, natural killer; TNF-α, tumor necrosis factor alpha; UMAP, uniform manifold approximation and projection.

Given the finding of activated NK cells with an ISG signature in acute HCV infection, we sought to validate these findings via flow cytometry in a larger cohort of patients with acute HCV and compared it to chronic HCV infection. Looking at frequency and phenotype of NK cell subsets, a shift toward more frequent CD56^bright^ NK cells and an elevated activation level could be observed in acute HCV compared to healthy controls and chronic HCV (Figure 2*A*–*C*, Supplementary Figure 2*A–D*). Of note, also in flow cytometric analysis, we observed a subset of activated NK cells in acute HCV, as marked by increased expression of Ki-67 and CXCR6 (Figure 2*A*–*C*, Supplementary Figure 2*D*). Matching the flow cytometric results to CITE-seq analysis, the acute HCV patients selected for CITE-seq had results similar to the overall cohort of acute HCV (Supplementary Figure 2*E*). In line with the flow cytometric findings, we identified reduced transcript expression of *FCGR3A* (CD16) and elevated *GZMB* as well as elevated CD38 on protein level (Figure 2*D*). However, these findings from CITE-seq analysis were more pronounced on the cell level than on the patient level (Figure 2*D*, Supplementary Figure 3*A*). Last, these alterations could also be confirmed in dimensionality reduction analysis of flow cytometric data where an enrichment of CD56^bright^ NK cells as well as granzyme B^high^ cells was observed in patients with acute HCV (Figure 2*E*).

Of note, while certain markers, such as granzyme B and CD38, could be identified both in transcriptome and via flow cytometry (Figure 2*B*, 2*D*, and 2*E*), Ki-67 and CXCR6 were only found via flow cytometry and not in CITE-seq analysis (Supplementary Figure 1*D*). Looking separately at CD56^bright^ and CD56^dim^ NK cells with flow cytometry, we observed that CD56^bright^ NK cells displayed elevated levels of activation/functionality markers (Supplementary Figure 3*B–E*). Hence, like the scRNA-seq data, NK cells displayed an activation profile within flow cytometric analysis.

As a last step, the phenotypic alterations were related to NK cell function by studying responsiveness to cytokines via interleukin (IL)–12/IL-18 stimulation and cytotoxicity via K562 stimulation (Supplementary Figure 3*F* and 3*G*). In line with the results from profiling the NK cell phenotype, we identified elevated degranulation and CCL4 (MIP-1β) production in response to cytokine stimulation and upon K562 stimulation in acute HCV (Figure 2*F*–*H*, Supplementary Figure 3*F* and 3*G*). These findings were predominantly in CD56^bright^ NK cells as no significant alterations could be observed in CD56^dim^ NK cells (Figure 2*F*–*H*, Supplementary Figure 3*H–J*).

Taken together, flow cytometry analysis confirmed the presence of an activated NK cell subset in acute HCV and could link phenotypic and transcriptomic changes to altered NK cell function.

### Heterogenic Origin of Activated NK Cell Subset in Acute HCV

**Figure 3.**
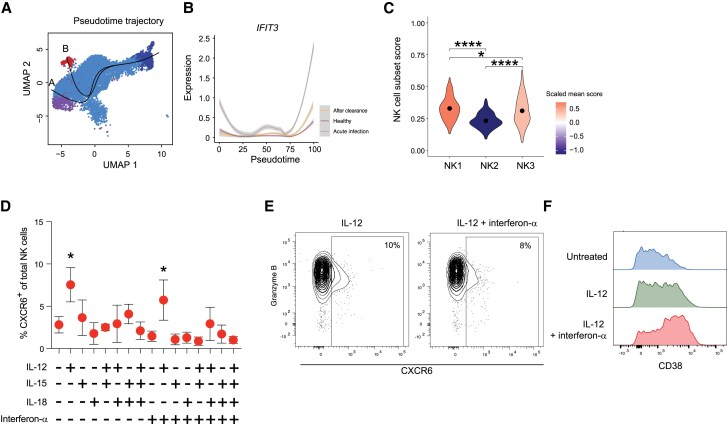
IL-12 and type I interferon as main driver for cluster of activated NK cells. *A*, Slingshot analysis of scRNA-seq data displayed on NK cell subsets identifying 2 trajectories. *B*, Representative expression of *IFIT3* along slingshot trajectories. *C*, NK cell subset scores (NK1, 2, and 3) on the identified activated NK cell subset. *D* and *E*, CXCR6 expression on enriched NK cells after 5-day incubation with the indicated cytokine combinations; displayed are summary (*D*) and representative (*E*) flow cytometry stainings. *F*, Representative CD38 expression on enriched NK cells after incubation with the indicated cytokines for 5 days. Statistically significant differences calculated in (*D*) with analysis of variance (comparison to untreated), *P < .05, in (*C*) *FDR <0.05 and ****FDR <0.0001. Abbreviations: FDR, false discovery rate; IL, interleukin; NK, natural killer; scRNA-seq, single-cell RNA sequencing; UMAP, uniform manifold approximation and projection.

Given the presence of a subset of activated NK cells in acute HCV, we next aimed to study the origin of these cells. To this end, we investigated the scRNA-seq data both via a pseudotime trajectory analysis and by calculating a gene set activity score for each cell based on the markers identifying the subsets NK1, 2, and 3 (Figure 3*A*–*C*). The cluster of activated NK cells displayed high scores predominantly for NK1 (Figure 3*C*, Supplementary Figure 4*A*). Along similar lines, the pseudotime trajectory also indicated a pathway from NK1 cells to the activated NK cells that was paralleled by increasing expression levels of *IFIT3* (Figure 3*B*). To elucidate the master regulatory genes and cytokines associated with this trajectory, we conducted a gene regulatory network analysis (regulons) using the single-cell regulatory network inference and clustering (SCENIC) workflow [17] and assessed cytokine signaling networks (Cytosig analysis [18]) within the identified NK cell clusters (Supplementary Figure 4*A* and 4*B*). In regulon analysis, the cluster of activated NK cells displayed elevated activity of cytokine/activation-related transcription factors *ETS2* and *E2F1* (Supplementary Figure 4*B*). Furthermore, Cytosig analysis revealed a clear IFN signature that could be detected in all NK cell subsets, albeit at higher levels in the activated NK cell cluster (Supplementary Figure 4*C*).

Given the significant enrichment of ISG-related transcripts and regulons/pathways indicating cytokine activation in the activated NK cells, we next investigated which cytokines might give rise to this population. Given the clear imprint of IFN in these cells, we stimulated isolated NK cells with type I IFN and combinations of IL-12, IL-15, and IL-18 as NK cell–related cytokines that have been shown to be elevated in acute HCV [19]. Of interest, type I IFN treatment alone only led to slight alterations (Figure 3*D* and 3*E*, Supplementary Figure 4*D*). A main driver for the elevated expression of CXCR6 was IL-12 (Figure 3*D* and 3*E*). However, while IL-12 alone had a minor impact on CD38 expression, combined stimulation with type I IFN and IL-12 led to a significant upregulation of CD38 and CXCR6 (Figure 3*D–F*, Supplementary Figure 4*D* and 4*E*).

In sum, these data indicate that type I IFN in combination with IL-12 are the key cytokines inducing activated NK cells in vitro recapitulating the phenotype observed in acute HCV.

### Normalization of NK Cell Activation Level During Treatment

**Figure 4.**
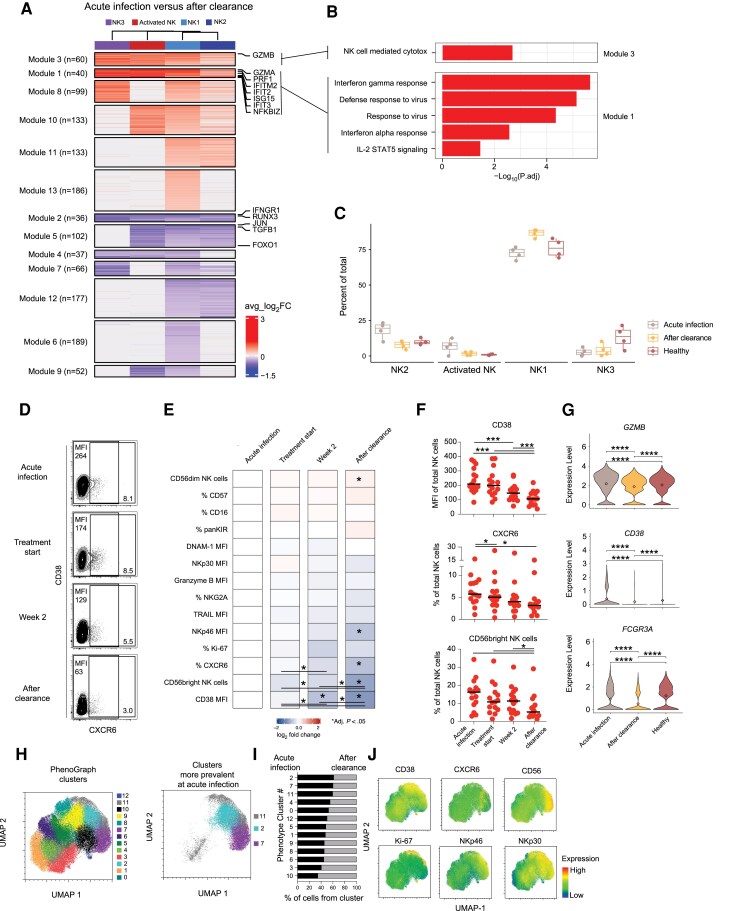
Normalization of NK cell compartment after direct-acting antiviral–mediated cure from acute HCV. *A–C*, Fold changes of DEGs (rows) in the indicated NK cell subsets (*A*), with insignificant values (adjusted *P* > .05) set to 0. Genes were grouped into modules through unsupervised k-means clustering. *B*, Bar plots displaying pathway enrichment for genes for the indicated modules. Red bars represent the upregulated pathways in patients during acute HCV infection. *C*, Comparison of the NK cell proportions between acute HCV infection, patients after viral clearance, and healthy individuals. *D–F*, Longitudinal flow cytometric assessment of NK cell phenotype at the time-points before, during, and after antiviral treatment (n = 15/15/15/14, respectively). Displayed are representative flow cytometry plots (*D*), summary data in relation to timepoint during acute infection (*E*), and scatter dot plots for CD56^bright^ NK cells (*F*) for the indicated markers. *G*, Representative expression levels from scRNA-seq data for the indicated genes. ****FDR <0.0001. *H–J*, UMAP and PhenoGraph analysis of pooled data from matched samples at the acute and viral clearance timepoints. PhenoGraph clusters are projected on the performed UMAP analysis (*H*); origin of cells was determined for each PhenoGraph cluster and the most frequent clusters during acute HCV infection (*I*). *J*, Selected parameters enriched in the presented markers in (*H*). Statistical analysis was performed by mixed-effects analysis followed by Holm–Sidak multiple comparisons test. *P < .05 and ***P < 0.001. Abbreviations: DEG, differentially expressed gene; FC, fold change; FDR, false discovery rate; HCV, hepatitis C virus; IL, interleukin; MFI, median fluorescence intensity; NK, natural killer; scRNA-seq, single-cell RNA sequencing; UMAP, uniform manifold approximation and projection.

Clinically, treatment with DAAs leads to a rapid drop in viral titers and subsequently to cure from HCV [4]. Thus, we had the chance to investigate if the observed alterations in the NK cell compartment in acute HCV infection are reversible upon rapid antiviral treatment. When analyzing longitudinal scRNA-seq data, a clear reduction of the infection imprint could be observed upon clearance. In detail, transcripts related to NK cell activation (*PRF1*, *IFIT2*, *NFKBIZ*) as well as pathways downstream from those (NK cell–mediated cytotoxicity) were found to be elevated during acute infection compared to resolved infection (Figure 4*A* and 4*B*). Of note, these alterations were present in most NK cell subsets (Figure 4*A*). Looking at the frequency of NK cell subsets in the scRNA-seq data, a shift from an elevated proportion of NK2 and activated NK cells during the acute infection at toward more frequent NK1 after clearance was observed (Figure 4*C*). These findings could also be recapitulated in flow cytometric analysis, as indicated by an increase in frequency of CD56^dim^ NK cells after clearance and a parallel reduction of CD56^bright^ NK cell frequencies (Figure 4*D*–*F*, Supplementary Figure 5*A*). In both flow cytometry and transcriptomic data, a reduction of NK cell activation throughout the antiviral treatment period was observed (Figure 4*E*–*J*, Supplementary Figure 5*A* and 5*B*). In detail, elevated expression of the transcripts *GZMB* and *CD38* paralleled by lower expression of *FCGR3A* (CD16) was observed in scRNA-seq analysis (Figure 4*G*). In line with this, analysis of clusters with PhenoGraph on flow cytometric data recapitulated these results as indicated by an enrichment of CD56^bright^ NK cells as well as CD38^high^ Ki-67^+^ NK cells at acute infection (Figure 4*H*–*J*, Supplementary Figure 5*C–E*). Finally, a degree of normalization of functionality could be seen. In CD56^bright^ NK cells, granzyme B levels dropped in response to IL-12/IL-18 stimulation after viral clearance whereas the response to K562 stimulation was comparable before and after viral clearance (Supplementary Figure 6*A–E*). No changes in CD56^dim^ NK cell functionality could be observed over time (Supplementary Figure 6*A–E*).

Overall, these data suggest that viral clearance by DAA treatment in acute HCV leads to rapid normalization of the NK cell phenotype and function.

### Persisting Imprint of HCV Infection on the NK Cell Compartment

Previously we have reported a persisting imprint on the NK cell compartment after cure of chronic HCV [20] as well as on the soluble immune milieu after clearance of acute HCV [19]. In the current study we had the opportunity to compare the effect after clearance of long-lasting chronic versus short acute infection.

**Figure 5.**
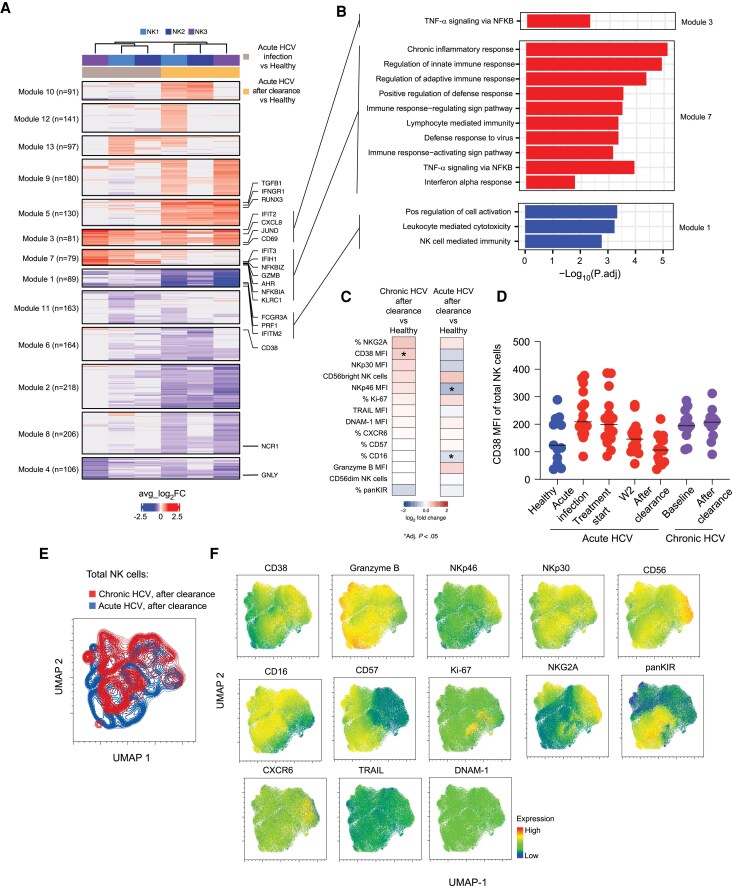
Persistent impact of acute HCV infection on NK cells after clearance. *A*, Identified clusters of differentially expressed genes stratified for NK cell subset and timepoint of sampling in comparison to healthy controls (n indicates the number of genes in the module). *B*, Pathway analysis of the indicated clusters from (*A*). *C*, Comparison of flow cytometry data from chronic (n = 12) and acute patients after viral clearance (n = 14) to healthy controls (n = 13). *D*, Representative data of CD38 MFI for the indicated cohorts. *E* and *F*, UMAP analysis on concatenated samples from patients with acute or chronic HCV after viral clearance. Displayed is overlay plot (*E*) and markers included in the analysis (*F*). Mann–Whitney test was used for comparison of acute and chronic samples after viral clearance/at follow-up, *P < .05. Abbreviations: FC, fold change; HCV, hepatitis C virus; MFI, median fluorescence intensity; NFKB, nuclear factor-κB; NK, natural killer; scRNA-seq, single-cell RNA sequencing; TNF-α, tumor necrosis factor alpha; UMAP, uniform manifold approximation and projection.

To address this, we determined first the impact of acute infection on the transcriptome of NK cell subsets compared to healthy controls. As the activated NK cell subset rapidly decreased during treatment, we focused on the major NK cell subsets, NK1–NK3 (Figure 5*A*). Of note, a module of enriched genes (module 7), marked by activation-related transcripts such as *IFIT3*, *GZMB*, and *NFKBIA* and deducted pathways “regulation of innate immune response and defense response to virus,” was elevated in NK1-3 and normalized from acute infection to clearance in comparison to healthy controls (Figure 5*A* and 5*B*). In contrast, a persistent imprint was seen regardless of the infection status. In detail, module 3 marked by elevated expression of activation-related transcripts such as *CD69* and *IFIT2* and positive enrichment for the pathway “TNF signaling via NFKB” was found in NK1–NK3 both during acute infection and at after clearance (Figure 5*A* and 5*B*). Furthermore, after clearance a persistently lower expression of *FCGR3A* and *PRF1* was observed along with a negative enrichment for NK cell–mediated immunity (Figure 5*A* and 5*B*).

Of interest, in comparison to healthy controls, chronic infection was marked by persistently elevated expression of CD38 after viral clearance in contrast to the significant decrease observed after clearance of acute HCV infection (Figure 5*C–E*, Supplementary Figure 6*F*). Both in manually gated as well as in uniform manifold approximation and projection analysis, these different trajectories could be observed, indicated by high CD38, NKp30, and NKp46 in chronic HCV infection after viral clearance (Figure 5*C–F*, Supplementary Figure 6*F*). Last, after acute infection clearance, certain changes remained in comparison to healthy controls that were not detected in chronic HCV—for example, reduced expression of NKp46 found both on the transcript (*NCR1*) and protein level (Figure 5*A* and 5*C*).

Collectively, while the activation of NK cells largely returns to normal after acute HCV infection, both acute and chronic infection can partly imprint the NK cell compartment more long-term.

## DISCUSSION

Ever since the introduction of IFN-free DAA treatment, both chronic and acute infection with HCV can be rapidly cured with only minor side effects [4]. NK cells are known to take part in the antiviral immune response [7] and are of special interest in the context of HCV infection [8]. On both the single-cell transcriptomic and protein levels, we observed altered composition and activation of the NK cell compartment, indicated by an increased expression of *CD38* and *GZMB* paralleled by a loss of *FCGR3A* in the transcriptome, while the proportions of CD56^bright^ NK cells and proliferating NK cells were increased in protein analysis. These alterations are in line with previous studies investigating NK cells during acute HCV in the pre-DAA era, as both elevation of CD56^bright^ NK cells [10, 21] and/or a decrease of CD56^dim^ NK cells [11, 21] have been described.

Furthermore, scRNA-seq analysis revealed a subset of activated NK cells displaying a strong ISG signature. In patients with chronic HCV, an elevated expression of ISGs has been reported [22], highlighting the importance of this cytokine in the antiviral immune response regardless of disease stage. The subset of activated NK cells that we identified displayed elevated expression of proinflammatory cytokines such as *TNF*, *CCL3*, and *CCL4*. Corroborating these findings, a higher production of CCL4 was observed from CD56^bright^ NK cells in functional analysis, suggesting a role for these cytokines and chemokines in the anti-HCV immunity. Via flow cytometry, we could identify a subset of CXCR6^+^CD38^high^ granzyme B^high^ NK cells that is likely the phenotypic counterpart of the activated NK cells observed in scRNA-seq. Of note, we could not identify elevation of CXCR6 transcripts in the subset of activated NK cells. However, this might be a result from higher patient numbers in flow cytometry analysis versus scRNA-seq and lower sensitivity of detecting the transcripts for CXCR6 in scRNA-seq.

In mouse models, CXCR6^+^ NK cells have been described to mediate antigen-specific responses against haptens [23] and in humans it has been reported as a marker for liver homing [24, 25]. Hence, the temporary rise of a CXCR6^+^ population observed here is of special interest as it can be speculated that this population would have the capacity to home to the liver and contribute to the antiviral immune response. Induction of CXCR6 on NK cells has been shown to occur after IL-12/IL-15 stimulation [26]. Thus, the systemic inflammatory profile in acute HCV might then induce CXCR6 on NK cells to promote liver homing. Our regulon analysis supports such reasoning since it points toward an origin by activation/cytokine stimulation and a robust imprint of IFNs was seen in cytokine signaling. Furthermore, we could show in vitro that predominantly the combination of IL-12 and IFN-α induces high levels of CD38 as well as an upregulation of CXCR6. As these cytokines have been shown to be elevated in acute HCV [19], we hypothesize that IL-12 and type I IFNs are the key drivers for NK cell activation in acute HCV and in that way induce a subset of activated NK cells with elevated functionality and a possible liver homing capability. For NK cells, a close link between cytomegalovirus (CMV) infection and appearance of memory-like NK cell expansions has been described [27]. However, in chronic HCV, no additional effect could be observed on the CMV-related NK cell expansions [28]. Although the present study on acute HCV infection did not study NK cell expansions in detail, our findings are in line with previous observations in chronic HCV from Malone et al [28]. The predominant changes induced by acute HCV were in the CD56^bright^ compartment and the rise of a CXCR6^+^ subset, while adaptive NK cells remained largely unaffected.

The activation of NK cells that we describe in acute HCV infection is comparable to other acute viral infections. For instance, in COVID-19 and in dengue virus infection, an upregulation of CD38 as well as Ki-67 has been observed in the acute phase of the infection [29, 30]. The more pronounced alterations of CD56^bright^ NK cells are also in line with other infections, presumably due to the higher responsiveness of CD56^bright^ NK cells to cytokines [7, 29, 30]. However, we here report a specific subset that is marked by an ISG signature. Future studies should elucidate whether this is a common feature in the antiviral immune response or rather that this is more specific to acute infection with HCV.

Last, we studied if and how the NK cell compartment normalizes after clearance of acute HCV infection and how this compares to chronic HCV infection. On both the transcriptomic and proteomic levels, we saw a clear reduction of activation markers and immune response pathways when comparing acute infection with postclearance timepoints in acute HCV infection, whereas in chronic HCV we observed only a partial restoration upon clearance. This is in line with previous studies where an imprint of chronic HCV has been shown both for NK cells [20] and for T cells [31, 32]. However, after clearance of the acute infection certain traits remained, as indicated by lower levels of NKp46 and CD16 after clearance of the infection. It has previously been reported that expression of CD16, and consequently antibody-dependent cytotoxicity (ADCC), is reduced during chronic HCV [33]. Thus, it can be speculated that the persistent loss of CD16 after clearance of acute HCV leads to a reduced capacity to perform ADCC.

Furthermore, also on the transcriptomic level, a partial restoration with persistent elevation of activation-related genes (*JUND*, *IFIT2*) after viral clearance in acute HCV was observed in the present study. Previously we could show that also the cytokine milieu is more long-term affected by acute HCV infection [19]. Hence, it can be speculated that even in the acute manifestation of HCV, a persistent alteration of the cytokine milieu and NK cell compartment can be induced.

This study has a number of limitations. Only small numbers of patients could be studied, and transcriptomic data could be generated only for 4 patients with acute HCV and are missing for chronic HCV. However, the obtained results from scRNA-seq in this study could be confirmed by phenotypic and functional analysis via flow cytometry. Furthermore, as the acute manifestation of HCV infection is such a rare event, we argue that the sample numbers are sufficient to address the posed research questions.

Collectively, we describe in this study that the acute HCV infection leads to a rise of an activated NK cell subset that is largely normalized after viral clearance. We show that IL-12 in combination with type I IFN is able to induce the observed phenotype, implicating this cytokine-driven activation to an important factor for mounting a successful antiviral immune response. Last, also after acute HCV infection clearance, a certain imprint on the NK cell compartment remains. These findings highlight the molecular basis of NK cell activation necessary for a successful antiviral immune response in acute HCV infection.

## Supplementary Material

jiaf654_Supplementary_Data

## Supplementary Data

Supplementary materials are available at The Journal of Infectious Diseases online (http://jid.oxfordjournals.org/). Supplementary materials consist of data provided by the author that are published to benefit the reader. The posted materials are not copyedited. The contents of all supplementary data are the sole responsibility of the authors. Questions or messages regarding errors should be addressed to the author.
