# Supplementary material for: Transient Interferon-Driven Natural Killer Cell Activation in Acute Hepatitis C
Source: J Infect Dis. 2025 Dec 29;233(6):e1330–40. doi: 10.1093/infdis/jiaf654 (PMC13271410; doi:10.1093/infdis/jiaf654)
Supplement: jiaf654_Supplementary_Data [file jiaf654_supplementary_data.docx]

**Supplementary Materials for**

**Transient interferon-driven NK cell activation in acute hepatitis C**

Benedikt Strunz, Qiuyao Zhan, Tanvi Khera, Julia Hengst, Marija Jankovic, Katja Deterding, Annika Niehrs, HepNet Acute HCV IV Study Group, Markus Cornberg, Cheng-Jian Xu, Heiner Wedemeyer, Niklas K. Björkström

**This file includes:**

**Table S1. Clinical information**

**Supplementary methods**

**Fig. S1.**

**Fig. S2.**

**Fig. S3.**

**Fig. S4.**

**Fig. S5.**

**Fig. S6.**

**Supplementary references**

**Supplementary tables**

|  | **Acute** | **Chronic** | **Healthy** |
| --- | --- | --- | --- |
| **ALT (U/L)** | 494 (42-1704) | 47 (25-273) | NA |
| **AST (U/L)** | 140.4 (36-1011) | 39 (18-104) | NA |
| **Bili (mmol/L)** | 27.3664 (8.552-215.5104) | 8 (4-18) | NA |
| **Lymphocytes (1000/uL)** | 2.108 (0.72-3.9852) | 1.85 (0.7-3.3) | NA |
| **VL (IU/mL)** | 34000 (1900-5600000) | 635000 (37000-1900000) | NA |
| **Age (y)** | 47 (23-62) | 52 (24-82) | 43 (30-50) |
| **Gender (m/f)** | 8/7 | 7/5 | 7/7 |

**Supplementary table 1. Clinical information**

Clinical parameters for included patients and controls. Displayed are either median with range or distribution (gender).

**Supplementary methods**

*Patient and control cohorts and experimentation*

Ethical permission for sample acquisition and analysis was granted by the responsible local ethical committee. Acute hepatitis C samples were collected at Hannover Medical University (MHH) and other participating centres in Germany. Peripheral blood from patients with acute symptomatic HCV infection was sampled at the timepoint of diagnosis (acute infection), at start of treatment, and weeks 2, 6, and after viral clearance at week 24 after start of treatment (denoted as after clearance). Chronic hepatitis C and control samples were collected at MHH. All samples were acquired after written and informed consent. Peripheral blood mononuclear monocytes (PBMC) were isolated via density-gradient centrifugation and cryopreserved in liquid nitrogen until experimentation. One healthy control was excluded due to an aberrant outlier phenotype as 70% of this individual’s NK cell compartment consisted of CD56^bright^ NK cells and an underlying confounder could not be excluded. One acute HCV sample collected after viral clearance was excluded due to bad sample quality and low cell numbers.

*Cellular-indexing of transcriptomes and epitopes by sequencing (CITE-seq)*

From four patients with acute HCV samples at acute infection and after viral clearance as well as samples from four healthy controls were used for simultaneous assessment of transcriptome and surface proteome. To this end, samples were thawed, stained with the Totalseq B antibody-mix (Biolegend). Staining was performed according to manufacturer’s protocol, modified by addition of a biotinylated CXCR6 antibody during staining and a secondary staining step with oligonucleotide-tagged streptavidin. Next, samples were subjected to single-cell RNA sequencing with the 10x Genomics kit for 3’ single-index gene expression version 3.1 according to manufacturer’s instructions. Sequencing was performed on a Nextseq2000, P3 v3 at the Bioinformatics and expression Analysis core facility at Karolinska Institutet, Huddinge.

*Analysis of CITE-seq data*

Raw reads for transcriptome and protein markers were aligned to the GRCh38 human reference genome. Cell barcodes and feature count matrices were created by Cell Ranger software version 6. The aggregated filtered RNA and protein count matrix was processed using the R package Seurat (v 4.4.0) [1]. Cells where over 20% of reads mapped to mitochondrial genes, cells with fewer than 300 genes or more than 3000 genes, and genes expressed in fewer than five cells were removed from the count matrix. scDblFinder (v1.10.0) [2] was used to detect heterotypic doublets and confirm singlets. Only singlets were kept for further downstream analysis. After quality control, the CITE-seq dataset contained a total of 86,798 cells, 25,271 genes, and 141 proteins. Cells from 12 individuals (4 healthy individuals, 4 patients sampled during acute infection and after clearance) were integrated using reciprocal PCA [3]. Subsequently, gene expression was log-normalized and scaled. Protein expression was normalized by CLR transformation. Next, PCA was performed on the integration-transformed expression matrix, and the first 30 PCs were used for k-nearest neighbors clustering by FindNeighbors and FindClusters functions, finally visualized by Uniform Manifold Approximation and Projection (UMAP). Cell clusters were further annotated by combining the results from the Azimuth package and the expression level of known cell marker genes and proteins. NK cells were further subclustered. The R package AUCell (v2.0.1) was applied to calculate the gene set score based on the top 20 NK subsets-specific markers published in the literature [4]. Protein markers further supported cluster annotations. Differential expression analysis was performed using the FindMarkers function with the Wilcoxon Rank-Sum test in Seurat. For each comparison, genes expressed in at least 10% of cells in the tested group and Bonferroni-corrected p < 0.05 were considered significant DEGs. The FindAllMarkers function was used to identify cluster marker genes by comparing cells in the cluster to the rest clusters. After identifying DEGs, the genes with BH correction less than 0.05 and an absolute ‘avg_log2FC’ value larger than 0.2 in at least one group were selected to perform the k-means clustering. If the gene was not significant in other groups, the values were set to 0. We performed k-means clustering with K=13 based on the ‘avg_log2FC’ values, which showed the clear gene modules. The enrichPA and enrichGO functions from R package clusterProfiler (v4.4.4) [5] were applied for overrepresented pathways and GO terms separately. In addition, enricher function was used based on the MsigDB H (hallmark gene sets) database to focus on the immune-related pathways. Pathways with BH correction less than 0.05 were regarded as significant. SCENIC (v1.3.0) [6] and pyscenic (v0.12.1) [7] were used to identify activated regulons in each subset of NK cells. The regulon activity was then scaled and centered before visualization using R package ComplexHeatmap. To identify transcriptional trajectories for NK cell subsets, we performed pseudotime inference using Slingshot (v2.10.0) [8]. The starting cluster of the trajectory was chosen as ‘CD56+ high NK’, while UMAP coordinates were used for trajectory construction**.** Comparison of responsiveness across NK cell subsets and conditions, we saved the Seurat object as cell ranger output using the ‘write10xCounts’ function. We then computed cytokine activities for individual cells using CytoSig [9], with the parameter ‘-signature 0’ to include 43 high-confidence cytokines. We got the median value for each cell type and each condition.

*Flow cytometry and CITE-seq*

PBMC were thawed, washed, stained for phenotyping, or plated for functional assessment. For phenotypic staining, the cells were stained in FACS Buffer (PBS +2% FCS +2mM EDTA) for 20 minutes at room temperature before fixation/permeabilization for 40 minutes and, if performed, intracellular staining for 30 minutes. UMAP analysis was performed in Flowjo v 10 with standard parameters. Two patients were excluded from the functional analysis due to too low cell recovery after stimulation.

Cellular-indexing of transcriptomes and epitopes by sequencing (CITE-seq) was performed on samples from four acute HCV patients during acute infection and after viral clearance as well as four healthy controls. In brief, samples were stained with the Totalseq B antibody-mix (Biolegend) and subjected to single-cell RNA sequencing with the 10x Genomics kit according to the manufacturer’s instruction. Samples were sequenced at the Bioinformatics and expression analysis core facility at Karolinska Institutet, Huddinge. For detailed description see supplementary methods.

*Statistical analysis of CITE-seq data*

All statistical programming and data visualization were performed using R (version 4.2.0). A paired two-tailed *t*-test was used to compare the cell proportion of one cluster between acute infection and after clearance. Bonferroni-corrected p less than 0.05 was considered to be statistically significant.

*Statistics on flow cytometric data*

Statistical analysis was performed either with R, version 4.0.5, or Graphpad prism version 8.3.0. For comparison of three or more groups, either Kruskal-Wallis test followed by Dunn’s test was applied for unpaired samples or when comparing multiple phenotypic parameters (when displaying summary fold change data) FDR adjusted p values of the Kruskal-Wallis tests were calculated. For comparing longitudinal paired samples, due to missing values a mixed-effects model followed by Holm-Sidak’s multiple comparisons test was applied. For comparison of two groups, Mann-Whitney U-Test was used for calculation of statistical significant differences.

**Supplemental figures**

**
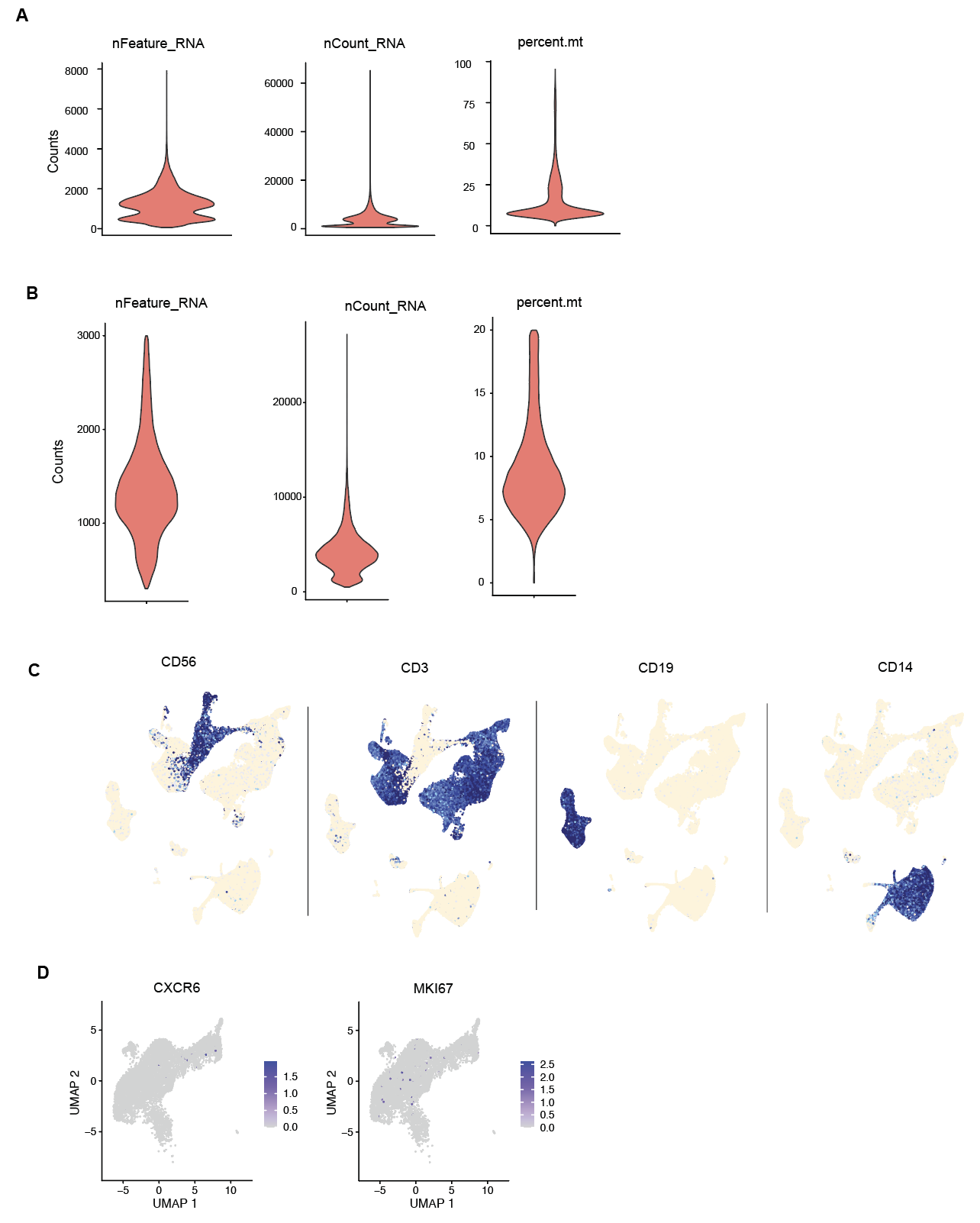
**

**Supplementary Figure 1. CITE-seq analysis of samples from acute and chronic HCV in comparison to healthy controls.**

(A and B) Violin plots displaying the distribution of three quality control metrics across single cells from all included samples (n=12) before (A) and after (B) applying filters. The nFeature_RNA represents the number of detected genes per cell, the nCount_RNA represents the total UMI counts per cell, the percent.mt represents the percentage of mitochondrial gene expression per cell. (C) Cell-type specific markers overlaid on UMAP embedding based on all cells, including CD56, CD3, CD19 and CD14. The UMAP plot is colored according to the protein expression levels. (D) UMAP plots displaying the expression of selected marker genes across NK cells. Each dot represents a single cell, and color corresponds to the normalized expression level of the indicated proteins.


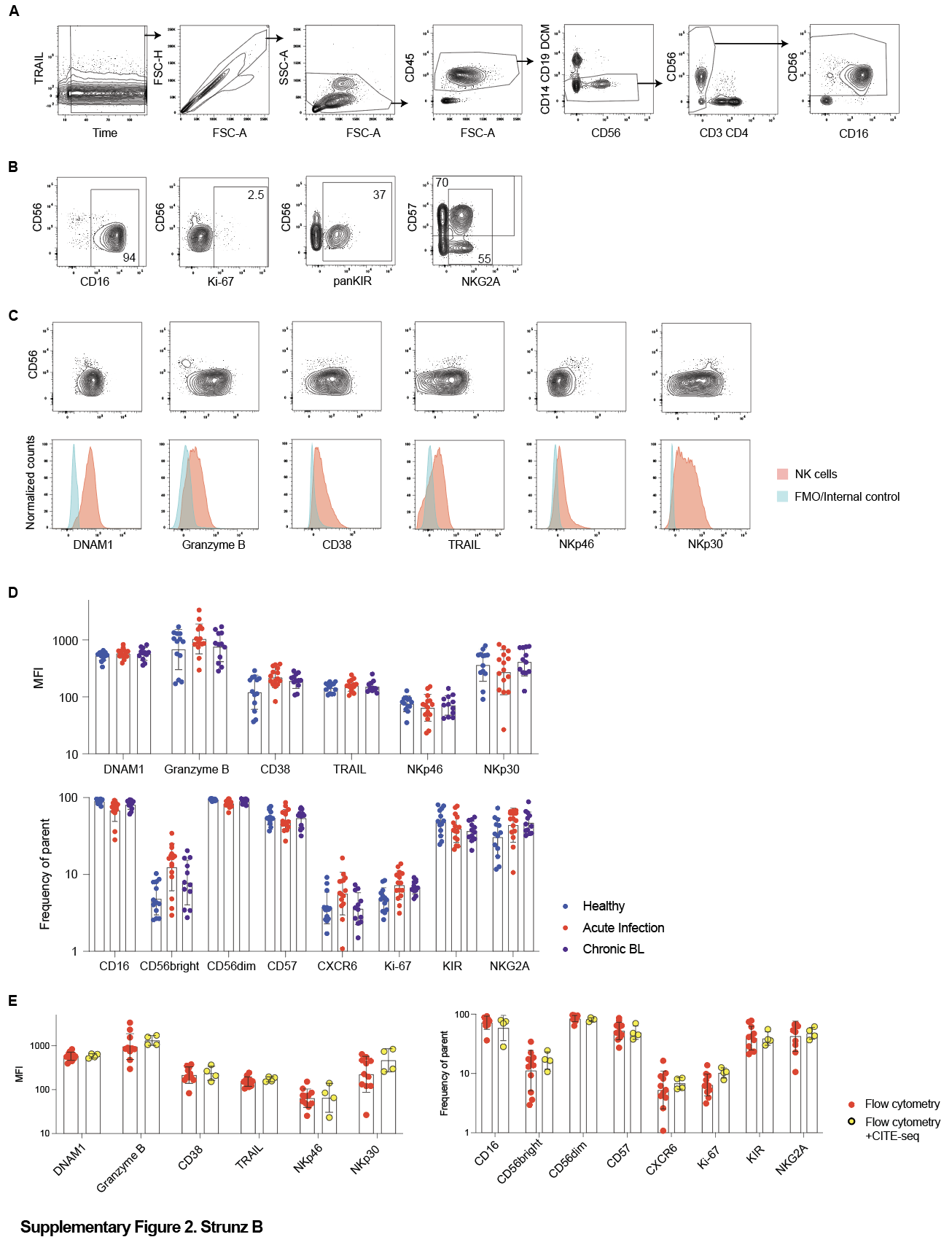


**Supplementary Figure 2. Phenotypic alterations in CD56^bright^ and CD5^6dim^ NK cells in acute and chronic HCV at baseline.**

(A) Representative gating strategy for identification of NK cells in flow cytometric analysis. (B and C) Representative flow cytometry plots for marker expression measured as frequency of parent (B) or marker expression as median fluorescent intensity (C, displayed as contour plot in upper row and as histogram plot with FMO or other internal control in lower row). (D) Display of indicated marker expression either as MFI (upper row) or frequeny (lower row) in the clinical groups. (E) Comparison of marker expression in acute HCV patients measured with only flow cytometry or flow cytometry and CITE-seq. Statistical significance was tested via Kruskal-Wallis test followed by Dunn’s multiple comparisons test. * indicates p<0.05.


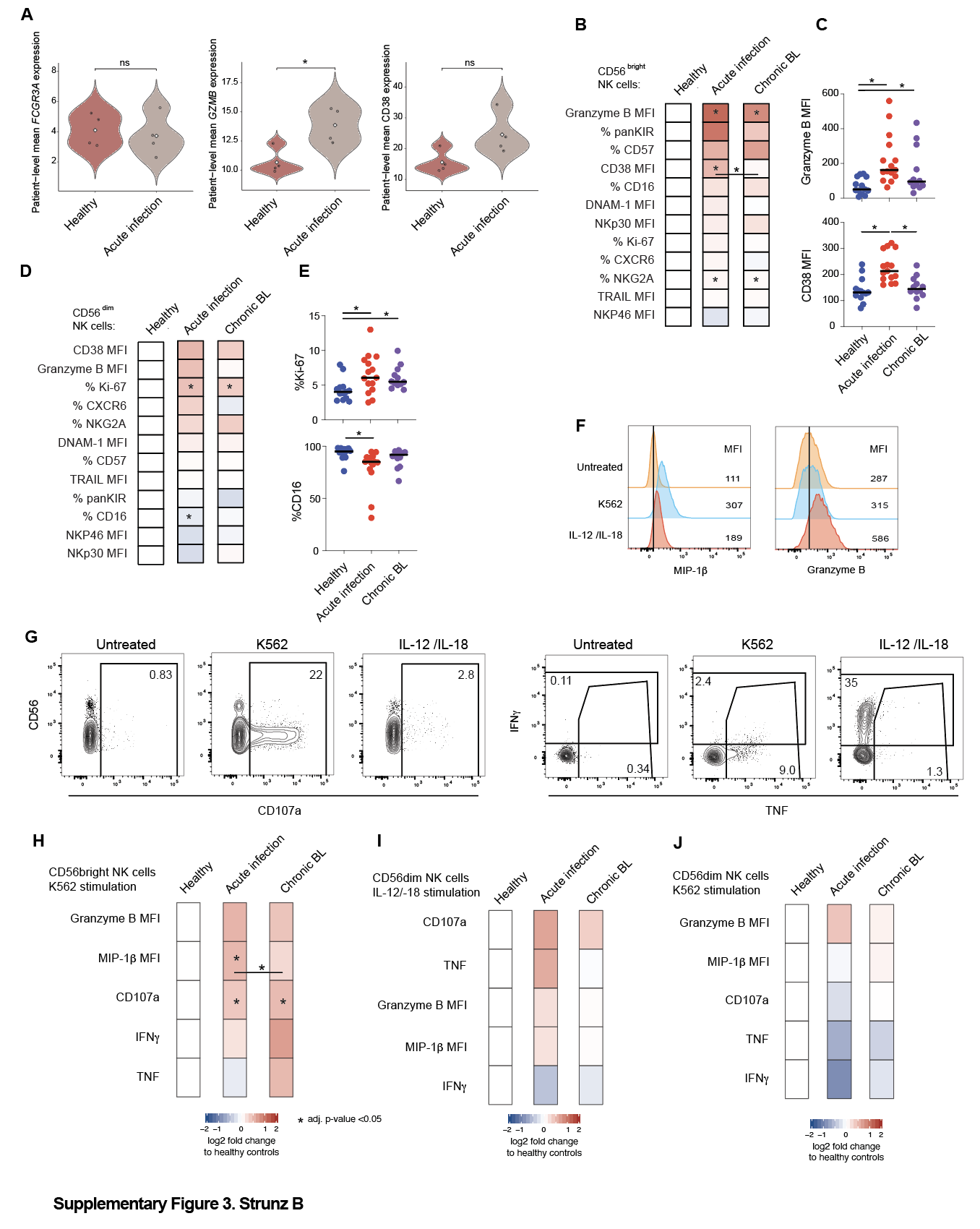


**Supplementary Figure 3. Alteration of NK cell phenotype and function occurs predominantly in acute HCV.**

(A) Expression of the indicated markers on patient-level as determined via CITE-seq, tested for statistical significant differences with Wilcoxon test. (B and D) Summary and representative data (C and E) of NK cell phenotype as determined with flow cytometry in CD56^bright^ (B and C) and CD56^dim^ (D and E) NK cells. (F and G) Representative flow cytometry plots displaying functional marker expression after the indicated stimulations either defined by change in MFI (F) or as frequency (G). (H-J) Function of CD56^bright^ (H) or CD56^dim^ (I and J) NK cells after stimulation with either K562 (H and J) or IL-12/IL-18 (I). (B, D, and H-J) Tested for statistical significance with Kruskal-Wallis test followed by Dunn’s test. * indicates p<0.05.


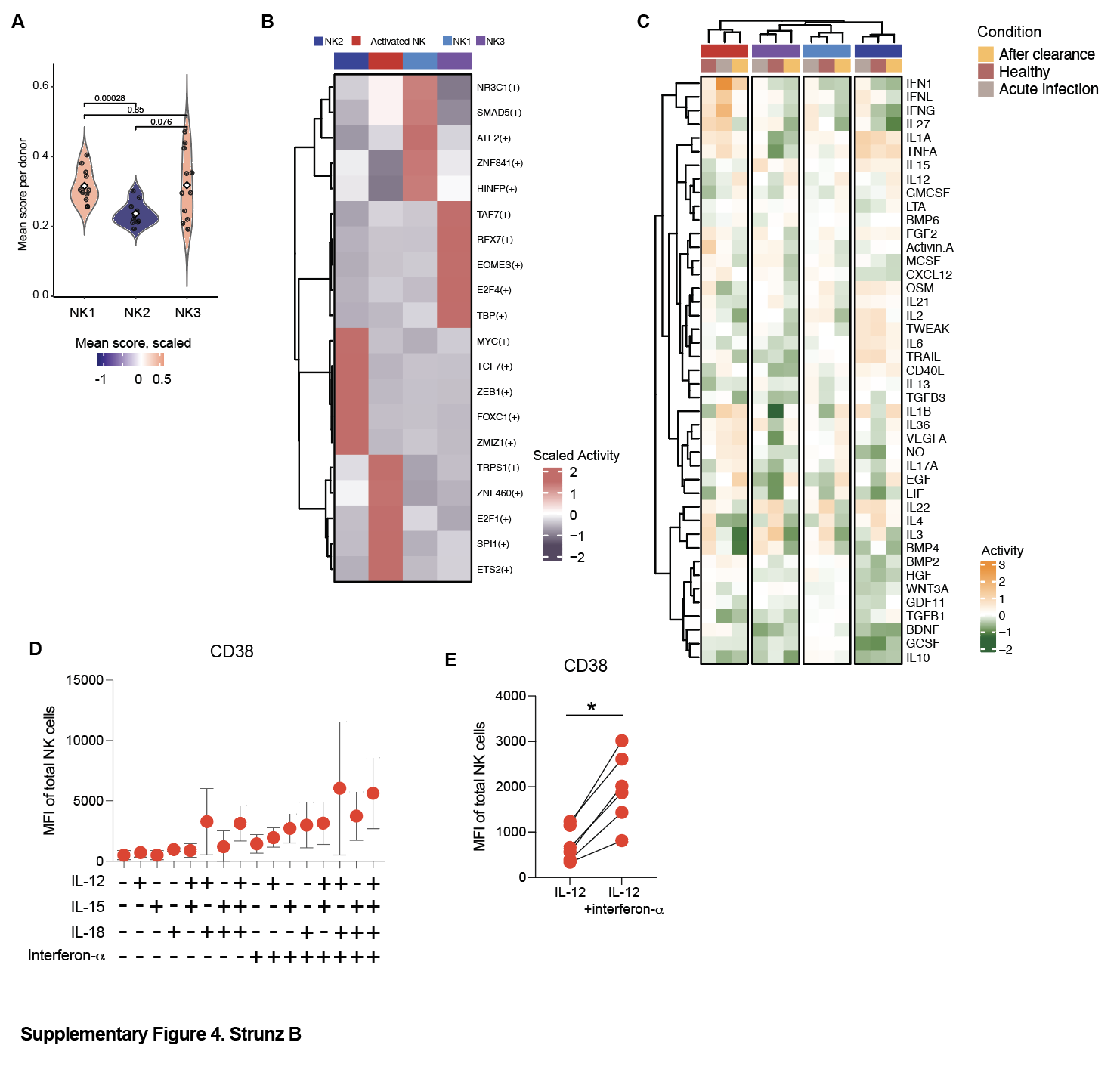


**Supplementary Figure 4. The regulome and cytokine signaling in NK cells in acute HCV and shaping of NK cell phenotype by cytokines.**

(A) Calculated NK cell scores on a donor level of the activated NK cell subset, Wilcoxon test was used for testing statistical significant differences. (B) Regulome of NK cells stratified for NK cell subset, analysed via SCENIC. (C) Cytosig-analysis of NK cell scRNAseq-data stratified for indicated condition. (D and E) Summary (D) and raw data (E) for expression of CD38 after incubation for five days with the indicated cytokine combinations, Mann-Whitney test was applied to test for statistical significance. * indicates p<0.05.

**
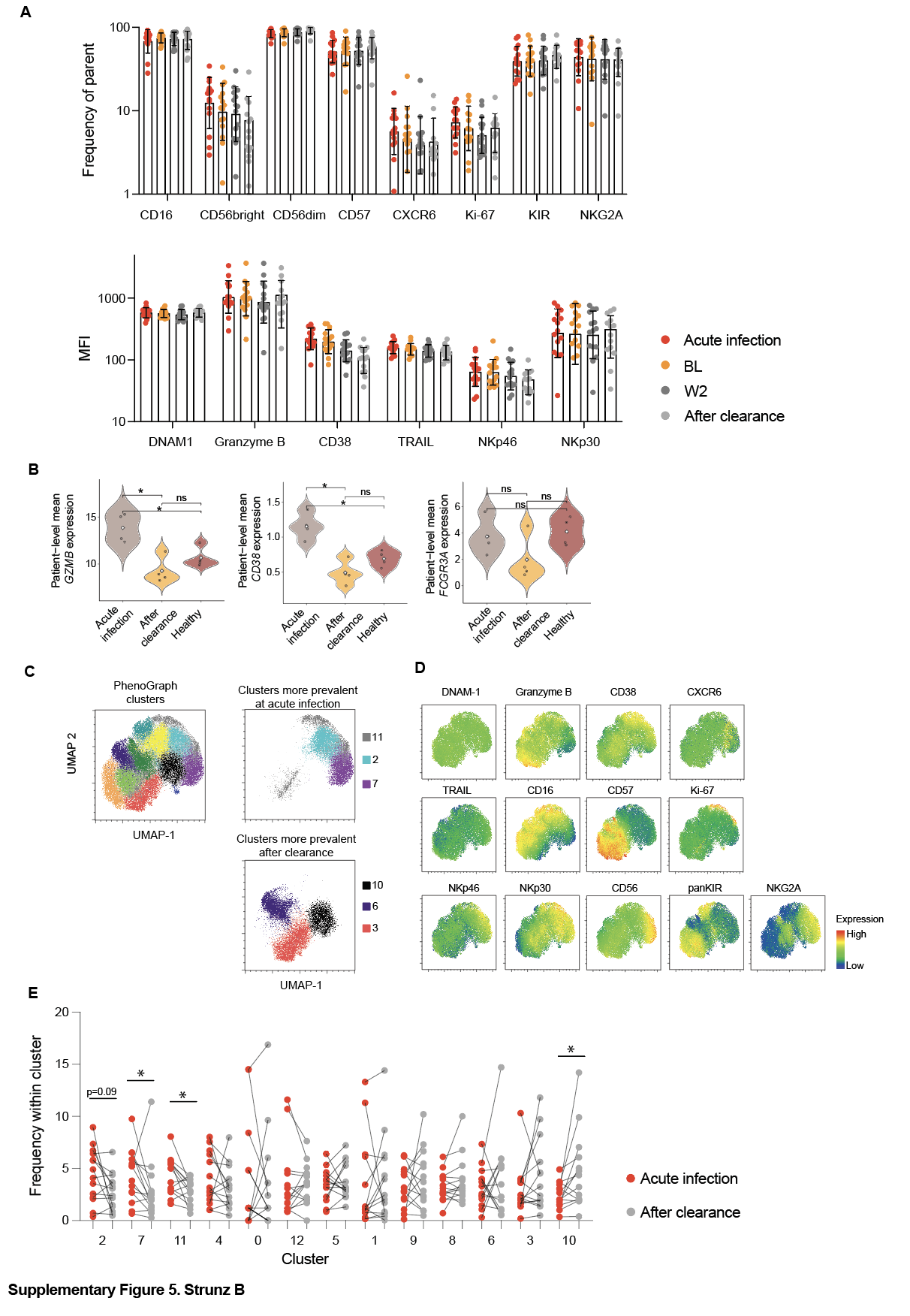
**

**Supplementary Figure 5. NK cell phenotype during and after clearance of HCV.**

(A) Marker expression in acute HCV patients at the indicated timepoints either determined as frequency (upper row) or MFI (lower row). (B) Expression of the indicated transcripts on patient level. (C and D) Complete PhenoGraph and UMAP data as displayed in Figure 4 (H-J). (C) PhenoGraph clusters calculated from flow cytometric data are overlayed on UMAP projection and (D) all markers included in UMAP. (E) Frequency of each individual donor of the in (C) displayed PhenoGraph cluster. Statistical significant differences were calculated with Wilcoxon test, * indicates p<0.05.


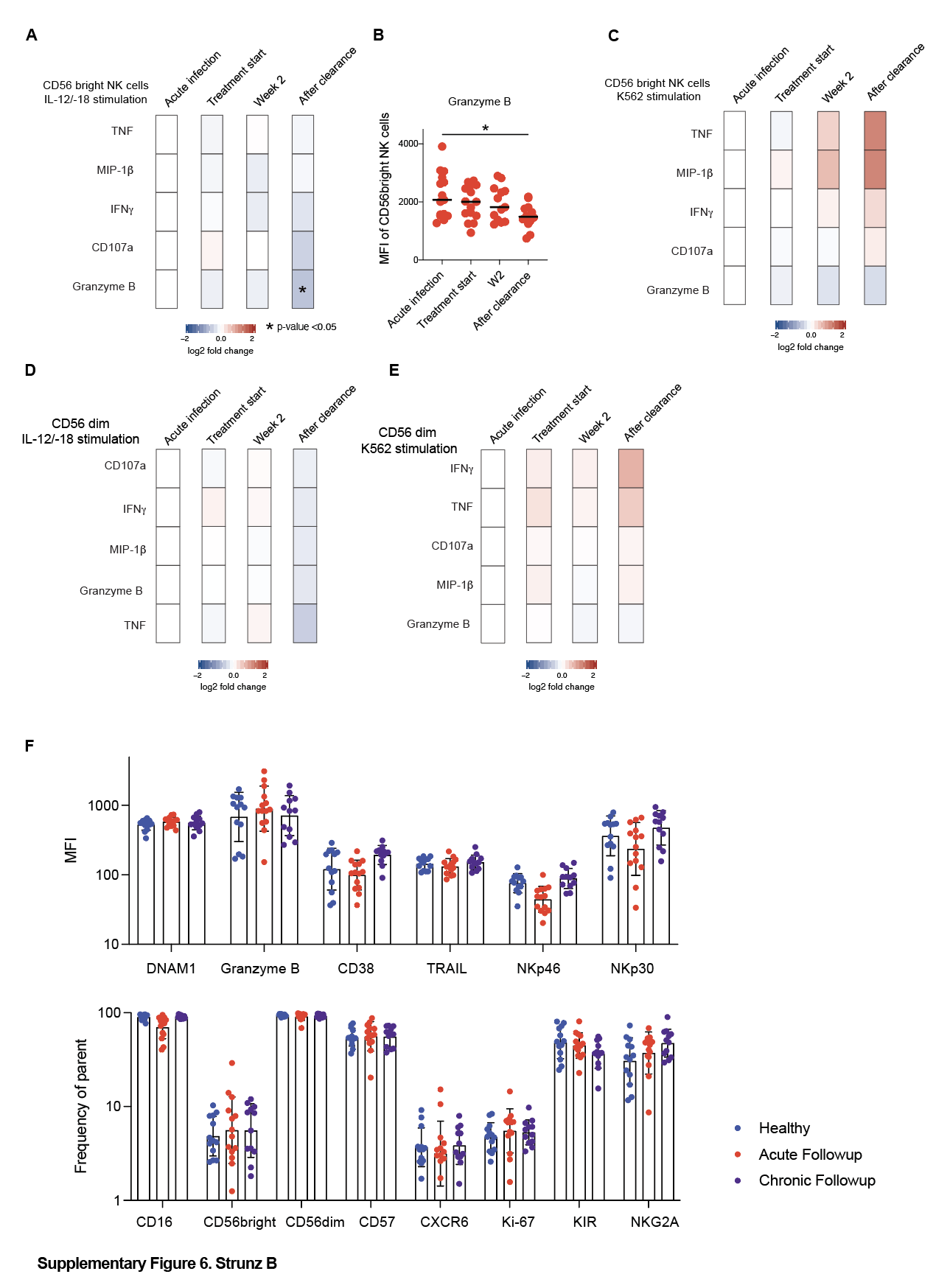


**Supplementary Figure 6. Modulation of NK cell function during treatment and comparison of phenotype to chronic HCV infection.**

(A-D) Functional analysis of either CD56^bright^ or CD56^dim^ NK cells after the indicated stimulation (K562 or IL-12/IL-18). (A and C-E) Displayed are summary of functional markers stratified for timepoint of sampling and (B) underlying raw data. Significant differences in functional data were tested for with mixed-effects analysis followed by Holm-Sidak’s multiple comparisons test. (F) Comparison of indicated NK cell markers in healthy controls as well as in acute and chronic HCV patients, upper row determined as MFI and lower row determined as percentage. * indicates p<0.05.

**Supplementary references**

[1] Hao Y, Hao S, Andersen-Nissen E, Mauck WM, Zheng S, Butler A, et al. Integrated analysis of multimodal single-cell data. Cell 2021;184:3573-3587.e29. <https://doi.org/10.1016/j.cell.2021.04.048>.

[2] Germain P-L, Lun A, Meixide CG, Macnair W, Robinson MD. Doublet identification in single-cell sequencing data using scDblFinder. F1000Research 2021;10:979. <https://doi.org/10.12688/f1000research.73600.2>.

[3] Stuart T, Butler A, Hoffman P, Hafemeister C, Papalexi E, Mauck WM, et al. Comprehensive Integration of Single-Cell Data. Cell 2019;177:1888-1902.e21. <https://doi.org/10.1016/j.cell.2019.05.031>.

[4] Rebuffet L, Melsen JE, Escalière B, Basurto-Lozada D, Bhandoola A, Björkström NK, et al. High-dimensional single-cell analysis of human natural killer cell heterogeneity. Nat Immunol 2024;25:1474–88. <https://doi.org/10.1038/s41590-024-01883-0>.

[5] Wu T, Hu E, Xu S, Chen M, Guo P, Dai Z, et al. clusterProfiler 4.0: A universal enrichment tool for interpreting omics data. Innov 2021;2:100141. <https://doi.org/10.1016/j.xinn.2021.100141>.

[6] Aibar S, González-Blas CB, Moerman T, Huynh-Thu VA, Imrichova H, Hulselmans G, et al. SCENIC: single-cell regulatory network inference and clustering. Nat Methods 2017;14:1083–6. <https://doi.org/10.1038/nmeth.4463>.

[7] Sande BV de, Flerin C, Davie K, Waegeneer MD, Hulselmans G, Aibar S, et al. A scalable SCENIC workflow for single-cell gene regulatory network analysis. Nat Protoc 2020;15:2247–76. <https://doi.org/10.1038/s41596-020-0336-2>.

[8] Street K, Risso D, Fletcher RB, Das D, Ngai J, Yosef N, et al. Slingshot: cell lineage and pseudotime inference for single-cell transcriptomics. Bmc Genomics 2018;19:477. <https://doi.org/10.1186/s12864-018-4772-0>.

[9] Jiang P, Zhang Y, Ru B, Yang Y, Vu T, Paul R, et al. Systematic investigation of cytokine signaling activity at the tissue and single-cell levels. Nat Methods 2021;18:1181–91. <https://doi.org/10.1038/s41592-021-01274-5>.
